# Indigenous Women and Their Nutrition During Pregnancy (the Mums and Bubs Deadly Diets Project): Protocol for a Co-designed mHealth Resource Development Study

**DOI:** 10.2196/45983

**Published:** 2023-07-06

**Authors:** Stephanie Gilbert, Rachel Irvine, Melissa D'or, Marc T P Adam, Clare E Collins, Rhonda Marriott, Megan Rollo, Roz Walker, Kym Rae

**Affiliations:** 1 Faculty of Humanities and Social Sciences University of Queensland Brisbane Australia; 2 Aboriginal and Torres Strait Islander Studies Unit University of Queensland Brisbane Australia; 3 Mater Research Institute Aubigny Place Brisbane Australia; 4 School of Information and Physical Science The University of Newcastle Newcastle Australia; 5 School of Health Sciences College of Health, Medicine and Wellbeing The University of Newcastle Newcastle Australia; 6 Food and Nutrition Research Program Hunter Medical Research Institute Newcastle Australia; 7 Ngangk Yira Institute for Change Murdoch University Perth Australia; 8 School of Population Health Faculty of Health Sciences Curtin University Perth Australia; 9 School of Indigenous Studies University of Western Australia Perth Australia; 10 School of Population and Global Health University of Western Australia Perth Australia; 11 Faculty of Medicine The University of Queensland Herston Australia

**Keywords:** co-design, community-based participatory research, mHealth, Aboriginal and Torres Strait Islander, maternal health, pregnancy, nutrition, Indigenous women, diet, health literacy, Indigenous, Indigenous people, mobile phone

## Abstract

**Background:**

Nutrition in pregnancy is pivotal to optimizing infant growth and maternal well-being. The factors affecting Indigenous people’s food and nutrition intake are complex with a history of colonization impacting the disproportionate effect of social determinants to this day. Literature regarding the dietary intake or dietary priorities of Indigenous women in Australia is scarce, with supportive, culturally appropriate resources developed for and with this group rare. Research suggests mobile health (mHealth) tools are effective in supporting health knowledge of Indigenous people and positive health behavior changes when designed and developed with the expertise of Indigenous communities.

**Objective:**

This study seeks to build the body of knowledge related to nutrition needs and priorities for Indigenous women in Australia during pregnancy. Further, this project team and its participants will co-design an mHealth digital tool to support these nutrition needs.

**Methods:**

The Mums and Bubs Deadly Diets study recruits Indigenous women and health care professionals who support Indigenous women during pregnancy into 2 phases. Phase 1 (predesign) uses a mixed methods convergent design using a biographical questionnaire and social or focus groups to inform phase 2 (generative). Phase 2 will use a participatory action research process during co-design workshops to iteratively develop the digital tool; the exact actions within a workshop will evolve according to the participant group decisions.

**Results:**

To date, this project has undertaken phase 1 focus groups at all Queensland sites, with New South Wales and Western Australia to begin in early to mid-2023. We have recruited 12 participants from Galangoor Duwalami, 18 participants from Carbal in Toowoomba, and 18 participants from Carbal in Warwick. We are expecting similar numbers of recruits in Western Australia and New South Wales. Participants have been both community members and health care professionals.

**Conclusions:**

This study is an iterative and adaptive research program that endeavors to develop real-world, impactful resources to support the nutrition needs and priorities of pregnant Indigenous women in Australia. This comprehensive project requires a combination of methods and methodologies to ensure Indigenous voices are heard at each stage and in all aspects of research output. The development of an mHealth resource for this cohort will provide a necessary bridge where there is often a gap in access to nutrition resources for women in pregnancy in Indigenous communities.

**International Registered Report Identifier (IRRID):**

DERR1-10.2196/45983

## Introduction

Pregnancy is an opportune time for promoting nutrition and health behaviors given the nutritional needs of rapid mental growth and development [1,2]. Inadequate intake of key nutrients during pregnancy can have lifelong effects on brain function and in childhood. It can lead to stunting and increased risk of chronic disease in adulthood, including obesity, cardiovascular, metabolic, and endocrine diseases [3]. Globally, it has been reported that women commonly find it challenging to meet the pregnancy nutrition targets [4]. In 2019, the National Health and Medical Research Council of Australia sought applications for a Targeted Call for Research that specifically addressed nutrition in Aboriginal and Torres Strait Islander Peoples with one of the identified areas identified for research being mothers and their children.

The factors affecting Aboriginal and Torres Strait Islander (hereinafter respectfully referred to as Indigenous) people’s food and nutrition intake are complex. Colonization of Australia by European settlers since the late 18th century has created a legacy of trauma and dispossession for many members of Indigenous communities [5]. Traditional cultural practices that included ceremony, language, and the hunting, fishing, and gathering of diverse and rich animal and plant food sources were banned or greatly restricted by the colonizers, which has continued to impact the health of Indigenous communities nationally to this day [6]. Food insecurity, often linked to the introduction of Western diets, limited access to traditional food sources, and the prohibitive expense of and lack of access to nutritious foods in many homeland communities is an ongoing concern for Indigenous communities [5]. Social determinants, including housing, education, economic status, and access to services all impact on health. Indigenous Australians are disproportionately affected by food insecurity and socioeconomic determinants that can contribute to unhealthy food patterns (ie, the affordability of fast food) and development of diet-related chronic diseases [7]. For instance, Indigenous adults in Australia experience type 2 diabetes at a rate 3.3 times higher than non-Indigenous adults, this increases further in remote and very remote areas [8]. Lee and Ride [9] also reported that nutrition-related chronic conditions (diabetes, cardiovascular, and kidney diseases) account for at least 75% of the mortality gap between Indigenous and non-Indigenous adults.

In Australia, very few studies have explored what Indigenous Australian pregnant women are consuming or the quality of their dietary intake [9,10]. Further, there is a dearth of information regarding nutritional knowledge during pregnancy for Indigenous women; particularly lacking is literature describing Indigenous Australians’ worldviews on their needs and priorities around pregnancy nutrition [11]. Lee and colleagues [10] published the first report on an Australian Indigenous cohort (n=56; the Gomeroi Gaaynggal cohort) exploring the nutritional adequacy of maternal dietary intake during pregnancy. When compared to the Australian Guide to Healthy Eating food groups serving recommendations, the analysis found that none of the women in this cohort met the recommended daily food group servings. In addition, of the 5 key nutrients for optimal reproductive health (folate, iron, calcium, zinc, and fiber), the nutrients with the highest percentage of pregnant women achieving the nutrition reference values were zinc (77.6%) and folate (68.9%), whereas iron was the lowest (1.7%) [10,11]. The authors recognized the low generalizability of the study, though called for urgent strategies to optimize the dietary patterns of pregnant women in this cohort. Iron, iodine, and folate are critical nutrients for growth and development, yet intakes are often reported as inadequate in general populations of pregnant women [11].

The use of digital tools such as mobile apps or websites to aid in knowledge sharing or tracking of health is defined as mobile health (mHealth). Often, Indigenous communities in Australia lack access to traditional health care, and mHealth has been successfully used to bridge this gap in some communities [12]. These instruments can be effective in supporting the health knowledge of Indigenous people and aid positive health behavior changes when designed and developed with the expertise of Indigenous communities [13]. A recent study of Aboriginal and Torres Strait Islander women aged 16 to 49 years found 89.2% owned a smartphone and 93.1% had access to the internet at home [14]. Furthermore, research suggests when development of mHealth tools is led by Indigenous peoples for inclusion of Indigenous participants, the outcomes are improved [12,15,16]. Though many existing mHealth options for Indigenous Australians focus on general health behaviors or mental health [13], to date, this form of health tool is currently underused for nutrition and pregnant women alike [15,17]. This project offers a unique opportunity for the co-design and development of an mHealth app or digital tool that enhances community members’ understanding of health issues and actively engages women in the planning and management of their own nutrition during pregnancy. As part of ensuring that this project is easily identified by Indigenous community members as a culturally safe project, the title “Mums and Bubs Deadly Diets” has been chosen. In Australian slang, the term *bub* is often used for baby, and *deadly* is well-known slang within Australian Indigenous communities to mean something is excellent, wonderful, and community-driven.

This project team seeks further understanding of the behaviors of Indigenous women who are pregnant across approximately 7 sites of Australia. A mixed methods biographical survey and social or focus groups in phase 1 deliver the predesign phases of intervention and behavior stage mapping, while co-design workshops in phase 2 will address the digital tool elements necessary for program production and implementation. Ultimately, the goal is to create a digital tool that supports the provision of nutritional information and access to resources Indigenous Australian women require during pregnancy and to operate within holistically embedded Indigenous research values and principles.

## Methods

### Aim

The aim is to further build the body of knowledge related to diet intake, food insecurity, and nutrition support needs, which may include behavior change strategies relevant for Indigenous women from diverse urban, rural, and remote communities in Australia. This team will co-design a specification document and with further funding, an mHealth app or digital tool to support the nutrition needs of Indigenous women during pregnancy.

### Study Design

This study comes from an Indigenous standpoint and is underpinned by critical self-reflexive practices that are aligned with a number of Indigenous research methods, including Aboriginal participatory action research [18]. The project will be undertaken in 2 phases that will use both mixed methods and co-design methodologies. The method builds on Sanders and Stappers [19] co-design framework, with a particular focus on the “predesign phase” (phase 1) and the “generative phase” (phase 2). Phase 1 will use a mixed method convergent design following an adapted shortened biographical narrative interpretive method (BNIM). The methods for phase 1 are based on the BNIM, which reconstructs data to understand a topic through 2 tracks of a participant’s perspective [20]. First, how they lived their life, and secondly, how they interpret the relevant events as told through their personal stories within the focus group.

Findings from the focus groups in phase 1 will inform phase 2. Phase 2 will use a participatory action research process during co-design workshops with participants. This phase adopted the Kaupapa Māori method for phase 2 workshops [21]. Kaupapa Māori clearly speaks to the goals of the co-design process. This method will be adapted as needed for the Indigenous Australian context but the concept of creating meaning together, enabling research by Indigenous peoples, for Indigenous peoples, and with Indigenous peoples is inherent within the Kaupapa Māori approach [21]. Due to the nature of this method (co-design) and the Aboriginal participatory action approach that is being undertaken, the exact actions within a workshop will evolve according to the participant groups’ direction.

### Governance and Ethics

#### Governance

Study governance includes the research team of both Indigenous and non-Indigenous academics from a broad cross-section of academic backgrounds (research team), the Indigenous Steering Committee (ISC), and partner organizations. The study has been closely developed with the Aboriginal community–controlled health sector, and the project is partnered with Galangoor Duwalami Primary Healthcare Service (Maryborough, Queensland), Carbal Medical Services (Toowoomba and Warwick, Queensland), Pius X Aboriginal Corporation (Moree, New South Wales), and Moorditj Koort in the Perth metropolitan area in Western Australia.

The investigatory team are committed to the concept of making meaning together and have developed the governance with the ISC, an integral part of the project. Each partner site has nominated members to the ISC who are recognized as having appropriate cultural, community, and nutritional knowledge to be representing their community. Additionally, Indigenous members of the research team are active members of the ISC and anticipate that this facilitates strong relationships across the project. The ISC will meet quarterly and have an active role in project management, data storage and retention, data analysis, publications, and dissemination.

#### Ethics Approval

The project has been approved by ethics committees aligned with the principles in the Australian Institute of Aboriginal and Torres Strait Islander Studies (AIATSIS) Code of Ethics for Aboriginal and Torres Strait Islander Research (2020) as follows: Mater Misericordiae (62512), University of Queensland (2021/HE002031), Aboriginal Health and Medical Research Ethics Committee of New South Wales (1966/22), and Western Australia Aboriginal Health Ethics Committee (HREC1102). Indigenous ways of knowing, practices, and cultural expressions are fully incorporated into the study methods as per the AIATSIS Code of Ethics for Aboriginal and Torres Strait Islander Research (2020) [22].

### Settings

This project is being undertaken across 3 states (Queensland, New South Wales, and Western Australia) to ensure that the project is broadly representative of the views and needs of Indigenous women across Australia. It is not feasible to work with every Aboriginal or Torres Strait Islander nation of Australia, but these sites were chosen for their diversity in cultural community, community sizes, socioeconomic status (measured by Socio-Economic Indexes for Australia; SEIFA), and rurality (measured by the Modified Monash Model). The average community SEIFA score is 1000, where SEIFA measures the relative level of socioeconomic disadvantage or advantage based on a range of community characteristics taken from the national census. As the index decreases from 1000, so too does the socioeconomic index; likewise, as it increases above 1000, the affluence of the community increases. It is worth noting that the SEIFA score shown for the communities in Table 1 is for an entire community or town and is only an indication for a region. To reflect the economic diversity of the participants, we will also be asking about their household income in our surveys. The Modified Monash Model scale from 1 to 7 defines the rurality of a community, whereby 1 is a major city and 7 is a very remote community [23]. Table 1 shows the characteristics of the sites for this study and highlights the diversity of population size, rurality, and socioeconomic status.

**Table 1:** Demographic characteristics of study locations.

Site			Primary cultural identification		Total population, n (significant urban area according to ABS^a^) [24]		Indigenous population [24], %		Modified Monash Model scale [23]		SEIFA^b^ score [25]
**Queensland**											
	Toowoomba: Carbal Aboriginal Medical Services	Jagera, Giabal, and Jarowair [26]		173,204		5		2		989	
	Warwick: Carbal Aboriginal Medical Services	Githabul [27]		12,294		8		4		976^c^	
	Maryborough: Galangoor Duwalami Primary Healthcare Service	Butchulla [28]		27,489		6.3		6		912	
**New South Wales**											
	Moree: Pius X Aboriginal Corporation	Kamilaroi [29]		9346		21.5		4		917	
**Western Australia**											
	Midland: Moorditj Koort	Whadjuk Noongar [30]		5972		5.3		1		900	
	Armadale: Boodjari Yorgas	Whadjuk Noongar [30]		79,602		2.5		1		994	
	Joondalup: Aboriginal Women’s Health Service	Whadjuk Noongar [30]		154,445		0.6		1		1078	
	Northbridge: Aboriginal Women’s Health Service	Whadjuk Noongar [30]		1307		0.8		1		1016	
	Cockburn: Moorditj Koort	Whadjuk Noongar [30]		104,473		1.5		1		1033	

^a^ABS: Australian Bureau of Statistics.

^b^SEIFA: Socio-Economic Indexes for Australia.

^c^SEIFA score available for Local Government Area.

### Participant Recruitment

Participant recruitment strategy has been determined in collaboration with each site. Each organization has prominently displayed posters (on their noticeboards and electronic sites) and has flyers available for potential participants, with a QR code included on both documents leading to detailed participant information and contact information for the research team if potential participants have any questions. Some participating sites, Carbal (Warwick) and Galangoor Duwalami (Maryborough), have indicated the study is an ideal activity for existing community groups within their organizations, including women’s groups, mother and baby groups, and Elder groups. In these instances, the research team has attended these organizations’ community groups in advance to deliver a short presentation to group members about the study and answer questions asked by potential participants. Following the research team presentation to the site and to potential participants, the site arranged a time for data collection with the group of participants who consented. All participants are advised that only deidentified data will be used for this project and are given a full explanation of how data will be stored safely.

Consent forms are signed by all participants upon arrival at the data collection activity following a further opportunity for participants to clarify any concerns of consent. In recognition of the commitment by participants to the study, participants involved in phase 1 and phase 2 are offered a gift or voucher to the value of Aus $50 (US $33.45) for each phase as advised by the partner organizations.

### Inclusion and Exclusion Criteria

This study is recruiting 2 different types of participants. We are specifically recruiting women who identify as having Aboriginal and Torres Strait Islander heritage. These women may either be currently pregnant or have raised children in their lifetime. The study also includes women who are older community members or Elders to ensure that experiences of all women in the community are represented. Additionally, health care professionals from Aboriginal community–controlled health organizations who support women’s health are being recruited. This participant group can include either male or female participants who may or may not identify as having Aboriginal and Torres Strait Islander heritage. Aside from health care professionals, this study will exclude male community members. The digital tool is designed to be used during pregnancy; therefore, men are not appropriate participants for the project.

### Approach

#### Overview

**Figure 1:**
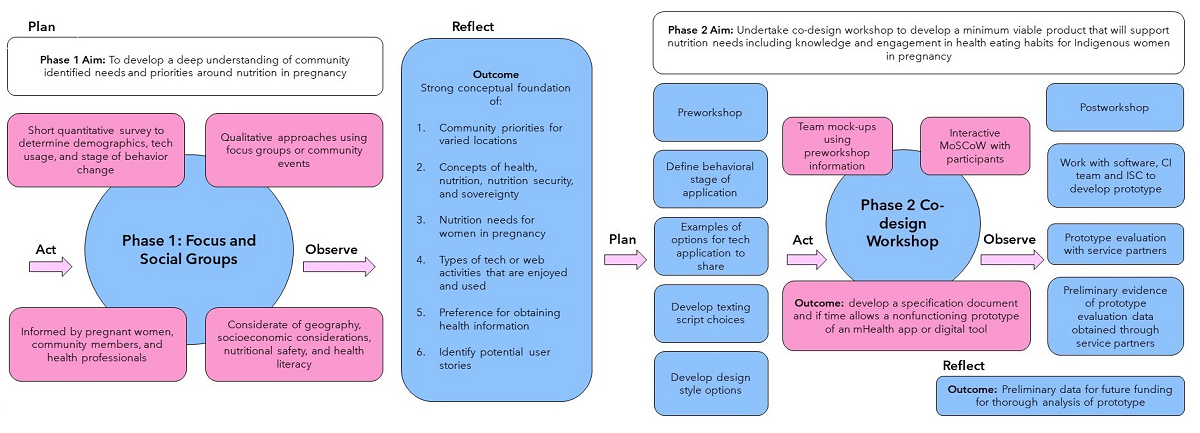
Co-design methods used and the integration of information from the phases 1 and 2 research cycle. CI: Chief Investigator; ISC: Indigenous Steering Committee; mHealth: mobile health; MoSCoW: must have, should have, could have, and would not have.

Phase 1 and phase 2 will occur as face-to-face discussions unless during the current pandemic health advice suggests otherwise. The project takes the holistic approach precedented by Chamberlain et al [31] using intervention mapping and action research cycle as shown in Figure 1. This study uses Aboriginal participatory action approaches discussed by Dudgeon et al [18] and also draws upon the guidelines to co-design mHealth systems developed by Noorbergen et al [32].

#### Phase 1: Understanding Community Worldview (Predesign Phase)

##### Overview

Focus and social groups will be held with at least 2 members of the research team. One member will be the primary group facilitator, and the other attendee will be there to assist with consent and survey paperwork. The other attendee will also take field notes during the discussions. The facilitator and other research team members will introduce themselves at the start of the focus group and spend some time talking informally to give participants time to relax prior to the start of the group.

**Figure 2:**
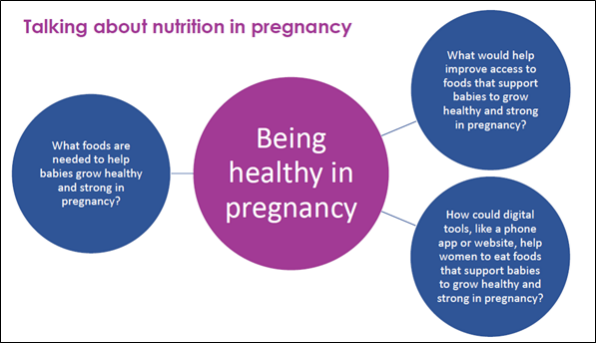
Social and focus group visual aid.

Focus groups with community members and health professionals will use similar prompt questions (Textbox 1) with the facilitator rephrasing these as appropriate for each group. To further aid discussion, visual aids are used to generate conversation during the social and focus groups and are placed around the room and to be used by participants throughout the discussion (Figure 2).

All participants attending will complete a short-answer survey designed by the research team. This will be completed at either the start or end of the group discussion. The survey contains standard demographic questions, current nutrition issues and food access experiences, and questions regarding current use of mobile phone apps, access to the internet, computers, and the types of activities used on the phone. When designing this survey, the team were aware that many research participants can feel that some questions have no relevance to the study. They made a conscious effort to only include questions that were directly related to the study outcomes and would be used in the data analysis for this study, and for which they could justify the use of the data to a participant who may query its purpose. Group discussions are between 1 and 2 hours depending on the group.

**Discussions with both women from the community and health care professionals are centered around the same prompts, reworded to suit their context.**

- What are the foods you or your community need to help babies grow healthy and strong in pregnancy?
- What would help you and your community to access foods that support babies to grow healthy and strong in pregnancy?
- How could digital resources, like a mobile phone app or a website, help you and your community to eat foods that support babies to grow healthy and strong in pregnancy?

##### Data Analysis

Survey data will primarily be assessed using descriptive statistics and later integrated with qualitative results in matrices. All focus groups are audio-recorded with participant permission and transcribed verbatim with thematic analysis undertaken. Transcripts and audio recordings are cross-checked for accuracy prior to analysis. Both deductive and inductive analyses will be undertaken. Analysis of the literature will provide a set coding framework (the deductive approach) that will be used across all sites and focus group transcripts by the research team. The inductive approach will develop site- or community-specific knowledge, while the deductive approach will provide a clear analysis framework that can be used for all sites that is specific to the objectives of the project. Inductive qualitative evaluation of the transcripts will use thematic techniques and insights to this cohort outlined by Sherriff et al [33] to guide the interpretation, thematic analyses, and reporting of this data. Using NVivo (version 12, 2018; QSR International Pty Ltd) qualitative data analysis software, identified themes will be compiled into a coding frame and, as new themes emerge, they will be compared against the initial coding frame and either added as new themes or used to expand and modify existing themes, until all data are accounted for.

The ISC will be enlisted to assist in the coding framework and contribute their understanding of any themes represented in the data using a chunk-by-chunk analysis format. Data analysis will be undertaken using constant comparison methods, and matrix displays will be used to explore similarities and differences across groups on key themes.

#### Prephase 2 (Generative Phase)

Data analysis of phase 1 will be completed from all sites prior to undertaking the phase 2 co-design workshops. Additionally, many resources to inform the co-design workshops will be compiled or developed by the research team based on the findings from phase 1.

Development of the workshop resources will occur in an active partnership with the ISC. Personas of potential users of the app will be determined and included in the developed presentation. The behavior stages of these potential personas will be identified to assess where the cohort’s understanding may be regarding a topic, ranging from precontemplation, contemplation, planning and preparation, action, and maintenance [34,35]. The team will compile initial concepts and themes for the app or tool and design or feature ideas to test and refine in co-design workshops with participant groups.

#### Phase 2: Co-design Workshop (Generative Phase)

##### Overview

Co-design workshops will be held with participants from phase 1 who can attend. However, it is expected that there will be a reduction in participant numbers as phase 2 workshops will take several hours. The start of co-design workshops will include additional introduction of new research team members attending and a short presentation of findings from phase 1 (likely delivered by the phase 1 facilitators). It is likely that this workshop will have 3 to 4 research team members attending throughout the workshop.

An additional interactive presentation will be delivered that will include mock-ups, ideas, and other information developed by the research team and the ISC during the prephase preparation. This will include a suite of technology probes (eg, screenshots of existing or relevant mHealth technology). The research team will discuss personas they have developed with the participants to consider if the target audience of the mHealth app or digital tool is appropriate. A facilitator from the research team will follow this with a 1-hour interactive discussion to build on feedback on these initial concepts with participants.

Regular breaks will be held throughout the workshop. Following a break, a further co-design activity will be undertaken with participants. This will be facilitated by one of the research team and it will include a strong focus on MoSCoW (must have, should have, could have, and would not have) activities [36]. This method is used in software development to assess how potential elements should be prioritized depending on participant opinion and is an ideal way of progressing from an idea through to the minimum viable product needed in mHealth product developments [36]. For a minimum viable product, such as the Mums and Bubs Deadly Diets project, the focus will be on the development of the “must haves” and “should haves.” Participants will be able to identify other desirable elements for the tool, with the research team ensuring that participants are aware of the timeline and budgetary considerations that can impact on the inclusion of these elements. In this case, elements will be identified in workshop activities such as a sticky notes exercise. Ideally, this workshop would have all participants and facilitators in 1 room throughout; however, with pandemic restrictions, the research team will be guided by health advice.

##### Data Analysis

Data collected in this phase includes audio recordings, transcripts, observer field notes as well as the collection of participant-developed workshop materials such as sticky notes and flip charts. The analysis of phase 2 is dependent on the outcomes of the co-design workshops and nature of workshop-developed materials. Using the aspects of interpretive phenomenological consensus analysis [37], the goal of this assessment is to identify exactly what these participants want in the project outcome. This may be the type of mobile technology, how it can be used, and the elements it contains, including nutrition knowledge and aspects related to health behavior change.

### Data Ownership and Management

Data management of this project has been undertaken to ensure that it reflects the 8 Indigenous Data Sovereignty principles identified by Trudgett et al [38]: ownership, control, accessibility, custodianship, accountability to Indigenous people, amplify the voice of the community, relevant and reciprocal, and sustainably self-determining. For the purposes of this study protocol, data refer to voice recordings, transcripts, survey responses, and files arising from the focus or interview recordings, including field notes and observations. The data arising directly from the participants of the study remain owned by the individuals; however, the analysis arising from the participants’ contributions is owned by the research team.

To ensure ownership and control, participants will have the option for their data to be handled in one of three ways after the project is complete, specifically 5 years after the final action of the project: (1) the data can be disposed of appropriately, (2) the survey and focus group data can be donated back to the community organization, or (3) all data can be donated to the AIATSIS data repository. This decision will be individual for survey data but must be unanimous for focus groups and workshops. Where a unanimous decision cannot be made, disposal of data will be the default. Whether data are returned to the community organization or AIATSIS, it will be sustainably held with Indigenous custodianship and appropriately accessible to the community.

During the life of the project, deidentified aggregate reports of initial findings will be shared with each site after each phase. These reports will be provided as an act of reciprocity to assist service strategic planning and amplify the voice of the participants.

### Dissemination of Results and Publications

This project seeks to follow the Consolidated Criteria for Strengthening the Reporting of Health Research Involving Indigenous Peoples (CONSIDER) statement [39]. This statement, developed through a meta-synthesis of international guidelines by a working group, identifies 8 key domains to guide the appropriate reporting of health research involving Indigenous peoples.

Results will be disseminated in several ways to meet the needs of the community and research team, including forums for the partner organizations and community members. Individual participants will be provided study outcomes and will also be encouraged to attend any public forums and research presentations. Publication of the outcomes from this work and this goal are embodied and enacted through the collaboration and Terms of References for the ISC and agreements made with partner organizations that include how the sharing and publication of material will occur.

## Results

To date, this project has undertaken phase 1 focus groups at all Queensland sites with New South Wales and Western Australia to begin in early to mid-2023. We have recruited 12 participants from Galangoor Duwalami, 18 participants from Carbal in Toowoomba, and 18 participants from Carbal in Warwick. We are expecting similar numbers of recruits in Western Australia and New South Wales. Participants have been both community members and health care professionals. Despite not asking specific questions about stages of behavior change, both community members and health professionals have raised this in a number of ways. Women and mothers have identified the things that they are currently practicing in their diets as well as areas where they are seeking further information and strategies, while health professionals have identified specific nutrition information that is challenging to explain related to pregnancy needs and highlighted the types of clients that would benefit and how best this information could be used in their practice. This will form part of the findings to inform phase 2. Preliminary findings have been disseminated to each of the Queensland sites as planned. Analysis is underway in preparation for phase 2.

## Discussion

### Principal Findings

This study has been designed using a combination of methodologies and methods to ensure such a major program is iterative, holistic, and culturally appropriate. A critical component to Indigenous research practice and more specifically the iterative nature of this project was reflexivity. This process encouraged shared understandings between the research team members and other collaborators, encouraging the positioning of Indigenous perspectives central to the project, allowing the research to remain ethical, meaningful, and useful throughout the many learnings and changes throughout the project [40].

The project takes a similar approach as that by Chamberlain et al [31] using intervention mapping and action research cycles to frame a co-design project from predesign and generation to implementation. Phase 1 will include the first 3 steps of intervention mapping: logic modeling of the problem, logic modeling of the change, and program design. Phase 2 will then address program production and some implementation concerns, though full implementation of evaluation will be addressed with future funding [41]. The action research cycle is used to intertwine the 2 phases in an iterative manner as highlighted in Figure 1. The dual cycle steps of “plan, act, observe, and reflect” among the 2 phases are linked, so the findings from surveys and group discussions of phase 1 inform phase 2.

The phase 1 biographical survey informs the lived life track through BNIM methods so that we may use the qualitative information to focus on the told stories track. Field notes and observations will be assessed to identify moments in the group conversation dynamics, linking transcripts to reality, emotions, and relationships. This process was adopted to allow this phase the in-depth understanding required to set the foundations for this project.

As highlighted by Noorbergen et al [32], prior to phase 2, the findings from phase 1 will have developed an intimate understanding for the research team of nutrition priorities for women during pregnancy. This will aid in determining what stages of behavior change the digital tool should focus on or may be added at each stage to support the journey of pregnant mothers. The behavioral stages identify where a person or cohort’s understanding may be regarding a topic [34,35]. This is an important component to designing the digital tool in a way that targets the correct level of comprehension and commitment of the target user. Although questions related to behavior change were not included in focus group guides, our preliminary analysis of phase 1 shows health professionals and community members have identified that they have distinct needs and varied stages of behavior change that they would like to address in the mHealth tool. This will encourage true engagement and retention with the information.

Prior to the workshops, the research team and the ISC will consider the types of user (persona) who will likely use this nutrition mHealth tool to ensure that each of these users is represented within its usage. For example, a technology tool focused on pregnancy nutrition for Indigenous women could be used by a woman during her pregnancy or used by a health professional to support health education during pregnancy. It will only be during the phase 1 that the team will be able to identify what needs are important for the community.

During phase 1, participants identify how they currently use their phones and consider how these could assist them with nutrition needs. These findings will assist in compilation of suitable technology approaches that can be shared, types of design options, as well as texting and script options that will be presented in phase 2 workshops. Feedback on probes will help to uncover types of technology and functions likely to be most effective in addressing the nutrition needs and priorities of Indigenous communities. The activities included are important to determine what aspects of the app must be standalone and what can be uploaded when an internet connection is available. It also helps with determining how diverse the activities within the app can or should be. For example, should it just contain written material, or are videos and pop quizzes more suitable to engage participants?

Kaupapa Māori concepts will be used throughout data analysis from phase 1 and during the co-design workshops in phase 2. This framework originally resonated with the research team due to these underpinning values that encapsulate the concepts of similar Indigenous Australian research concepts including Aboriginal participatory action research methods [18,42]. Embedding Kaupapa Māori principles positions the project within a research paradigm of epistemic self-determination. Indigenous Australian values, knowledge, and voices influence all aspects of the project.

Phase 2 has specifically been identified to require more research team members present, as differing areas of expertise will be required on the day. To ensure best outcomes from the workshops, all efforts will be made by the research team to relax participants, so that participants feel comfortable and confident to voice their opinions throughout. Breaks will no doubt be needed; however, they serve the dual purpose of people chatting informally and getting to know one another, which will aid in participation from all.

### Strengths and Limitations

The study’s priority is to inform and co-design a culturally appropriate digital tool to support nutrition needs of Indigenous women in pregnancy. The strength of this study is the co-design methods and approaches to “making meaning together” with Indigenous governance and partnered communities across the country. The consolidation of the co-design method, the development of an Indigenous governance structure, and the research team comprising Indigenous researchers and leaders ensure the study is culturally safe.

As a priority, this study endeavors to include sites across Australia in order to truly include the end users in the development of this tool and seeks to be respectful of Indigenous communities in Australia and their incredibly diverse values, traditions, and customs [12,16,43]. It is not possible to include every single Indigenous country of Australia (there are over 250 individual Aboriginal and Torres Strait Islander nations contained within Australia); therefore, these results may not be generalizable to all Indigenous Australian women. However, the spread across rural, coastal, and metropolitan communities and cultural groups will provide diversity to develop the mHealth product.

The COVID-19 pandemic continues to be a distinct limitation for this project. Local lockdowns and restrictions meant delays to project timelines, and significant modifications have been made to community engagement plans. Initial engagement meetings were moved to web-based platforms, which limited the ability to develop rapport with partner organizations and delayed the start of the study. Further, many community members have reduced their attendance to organized programs such as mothers groups, creating early challenges to recruitment. However, as we move further away from the major pandemic waves, we hope that COVID-19-related challenges are reduced.

The predicted users of this tool would be pregnant Indigenous women and potentially their health care professionals. As such, we felt it reasonable to limit our cohort to these 2 groups. However, participants have mentioned they would like their male partner or their children’s fathers to be engaged in the conversation as they may rely on them to do nutrition tasks within the home. The restriction of the study cohort to women may be a limitation of this study; however, they are the target intended users. Future research could consider including the entire family unit in these conversations.

### Conclusions

The Mums and Bubs Deadly Diets study is an iterative and adaptive research program that endeavors to develop real-world, impactful resources to support the nutrition needs and priorities of pregnant Indigenous women in Australia. This project addresses a problem from beginning to end, which requires a combination of methods and methodologies to ensure Indigenous voices are heard at each stage and in all aspects of research output. A mixed methods biographical survey and social or focus groups in phase 1 will deliver the predesign phases of intervention mapping, while co-design workshops in phase 2 will address the digital tool elements necessary for program production and implementation. The development of an mHealth resource for this cohort will provide an essential bridge, where there is often a gap in access to nutrition resources for women in pregnancy in Indigenous communities.

## Abbreviations

AIATSIS: Australian Institute of Aboriginal and Torres Strait Islander Studies
BNIM: biographical narrative interpretive method
ISC: Indigenous Steering Committee
mHealth: mobile health
MoSCoW: must have, should have, could have, and would not have
SEIFA: Socio-Economic Indexes for Australia

## Data Availability

In line with ethics approval, the data collected as a part of this study will not be publicly available. However, the data may be made available upon request for specific purposes to the corresponding author and with the agreement of the Indigenous Steering Committee.
